# *In vivo* characterisation of a therapeutically relevant self‐assembling ^18^F‐labelled β‐sheet forming peptide and its hydrogel using positron emission tomography

**DOI:** 10.1002/jlcr.3534

**Published:** 2017-08-04

**Authors:** O. Morris, M.A. Elsawy, M. Fairclough, K.J. Williams, A. Mcmahon, J. Grigg, D. Forster, A.F. Miller, A. Saiani, C. Prenant

**Affiliations:** ^1^ Wolfson Molecular Imaging Centre The University of Manchester UK; ^2^ CRUK/EPSRC Imaging Centre in Cambridge & Manchester The University of Manchester UK; ^3^ School of Materials The University of Manchester UK; ^4^ Manchester Institute of Biotechnology The University of Manchester UK; ^5^ School of Pharmacy and Biomedical Sciences University of Central Lancashire UK; ^6^ Manchester Pharmacy School The University of Manchester UK; ^7^ GE Healthcare Little Chalfont UK; ^8^ School of Chemical Engineering and Analytical Science The University of Manchester UK

## Abstract

Positron emission tomography (PET) and fluorescence labelling have been used to assess the pharmacokinetics, biodistribution and eventual fate of a hydrogel‐forming nonapeptide, FEFKFEFKK (F9), in healthy mice, using ^18^F‐labelled and fluorescein isothiocyanate (FITC)‐labelled F9 analogues. F9 was site‐specifically radiolabelled with 2‐[^18^F]fluoro‐3‐pyridinecarboxaldehyde ([^18^F]FPCA) via oxime bond formation. [^18^F]FPCA‐F9 in vivo fate was evaluated both as a solution, following intravenous administration, and as a hydrogel when subcutaneously injected. The behaviour of FITC‐F9 hydrogel was assessed following subcutaneous injection. [^18^F]FPCA‐F9 demonstrated high plasma stability and primarily renal excretion; [^18^F]FPCA‐F9 when in solution and injected into the bloodstream displayed prompt bladder uptake (53.4 ± 16.6 SUV at 20 minutes postinjection) and rapid renal excretion, whereas [^18^F]FPCA‐F9 hydrogel, formed by co‐assembly of [^18^F]FPCA‐F9 monomer with unfunctionalised F9 peptide and injected subcutaneously, showed gradual bladder accumulation of hydrogel fragments (3.8 ± 0.4 SUV at 20 minutes postinjection), resulting in slower renal excretion. Gradual disaggregation of the F9 hydrogel from the site of injection was monitored using FITC‐F9 hydrogel in healthy mice (60 ± 3 over 96 hours), indicating a biological half‐life between 1 and 4 days. The in vivo characterisation of F9, both as a gel and a solution, highlights its potential as a biomaterial.

## 1 INTRODUCTION

The potential of peptide hydrogels as biomaterials is vast on account of their *in vivo* and *in vitro* potential uses in a variety of biomedical applications. These include their use as scaffolding for 3D cell cultures, tissue engineering and regenerative medicine as well as their use as vehicle for localised drug delivery.^1^, ^2^, ^3^, ^4^, ^5^, ^6^ Peptide hydrogels are made of short synthetic peptides composed of either natural or nonnatural amino acids that are designed to self‐assemble into higher biomimetic structures. This is achieved via noncovalent interactions in response to external triggers, such as pH, ionic strength, enzymes and light, to form 3‐dimensional networks of entangled fibrous structures.^7^, ^8^

In the present study, the *in vivo* behaviour of a β‐sheet forming peptide FEFKFEFKK (F9) (F: phenylalanine; K: Lysine; E: glutamic acid) and its hydrogel was characterised. F9 is one of a family of amphipathic peptides with alternating hydrophobic and hydrophilic residues that self‐assembles into β‐sheet fibres that above a critical gelation concentration (CGC) associates to form a hydrogel. The properties of the hydrogels can be tailored through peptide design, concentration, media pH and ionic strength^9^; as such, they have been shown to hold promise for application in the biomedical field as 3D cell culture matrices^10^ and/or drug delivery vehicles.^5^

Positron emission tomography (PET) is a quantitative imaging modality boasting high levels of sensitivity. The tool was used to assess the behaviour of radiolabelled F9 ([^18^F]FPCA‐F9) *in vivo* using C3H mice. [^18^F]FPCA‐F9 was first delivered as a solution (concentration below CGC, pH 5.6) into the tail‐vein to study the fate of peptide monomer in the blood stream. Subsequently, [^18^F]FPCA‐F9 was delivered as part of an F9‐hydrogel (concentration above CGC, pH 5.6) by co‐assembly with unfunctionalised F9 peptide, before subcutaneous injection into the mouse flank.

**Figure 1:**
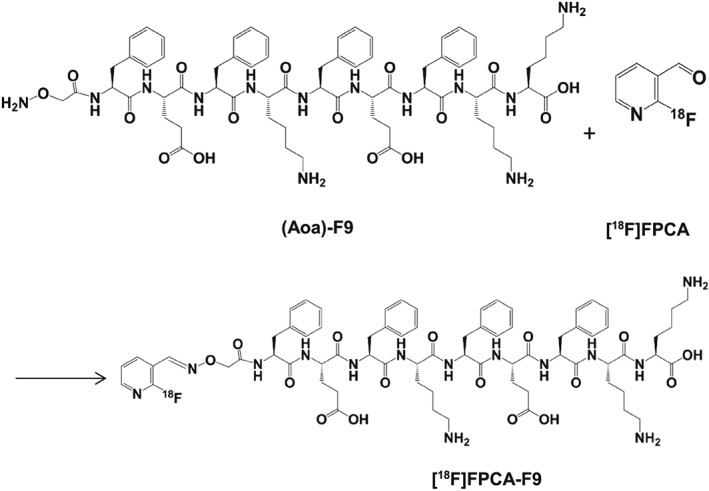
Reaction pathway showing (Aoa)F9 radiolabelling with [^18^F]FPCA

Site‐specific radiolabelling of F9 was selected to safeguard the β‐sheet forming and therefore the gel‐forming capability of the peptide. To achieve this, the *N*‐terminal amine was functionalised with an amino(oxy)‐group to give (Aoa)F9 (Mr 1338.59 Da). Functionalisation of the peptide with the amino(oxy)‐moiety permits its radiolabelling with the ^18^F‐prosthetic group 2‐[^18^F]fluoro‐3‐pyridinecarboxaldehyde (FPCA) via oxime‐bond formation, as has previously been described.^11^ The radiosynthesis pathway of [^18^F]FPCA‐F9 by radiolabelling of (Aoa)F9 with [^18^F]FPCA is shown in Figure 1.

PET studies were complemented by *in vivo* fluorescence imaging, making use of a fluorescein isothiocyanate *N*‐terminal labelled F9 peptide [fluorescein isothiocyanate (FITC)‐F9]. Fluorescence data provided information regarding the long‐term stability of the hydrogel, in healthy mice, over 5 days, a time‐frame outside the window of the radionuclidic half‐life of ^18^F (110 minutes).

## 2 EXPERIMENTAL

All solvents were purchased from Sigma‐Aldrich and used without further purification. (Aoa)F9 was purchased from Biomatik (Ontario, Canada) in trifluoroacetic acid (TFA) salt, purity >98%. FITC‐F9 was purchased from Biomatik as a TFA salt (purity >98%) and converted into the HCl salt of the peptide. [^18^F]Fluoride was produced onsite via the ^18^O(*p, n*)^18^F nuclear reaction by 16.4 MeV proton bombardment of enriched [^18^O]H_2_O using a GE PETtrace cyclotron (GE Healthcare, Sweden). Analytical HPLC was performed using a Shimadzu (Milton Keynes, UK) Prominence system (LC‐20AB solvent delivery system, SPD‐20A dual wavelength absorbance detector) controlled by LabLogic (Sheffield, UK), Laura 3 software via a CBM‐20A controller. HPLC eluate was measured for radioactivity using a Bioscan (Oxford, UK) Flow‐count B‐FC 3100 gamma detector. All preclinical PET scans were carried out using a Siemens (Oxford, UK) Inveon® PET‐CT scanner. Fluorescence *in vivo* imaging was carried out using a Biospace Lab Photon Imager Optima (Nesles‐la‐Valee, France) controlled by Biospace Lab Photo Acquisition software v3.4 and images analysed using Biospace Lab M3 Vision software.

## 3 RADIOCHEMISTRY

### 3.1 [^18^F]Potassium fluoride

Cyclotron produced [^18^F]fluoride was delivered to a TRACERlab FX‐FN radiochemistry system (GE Healthcare, UK) and trapped on a Sep‐Pak QMA cartridge (Oasis, Waters, UK). The cartridge was then eluted with a K_2_CO_3_ solution (0.01 M, 0.4 mL) into a reactor containing 18‐crown‐6 (8 mg, 30 μmol) in acetonitrile (0.6 mL). The mixture was azeotropically dried with 3 sequential additions of acetonitrile (1.6 mL total) at 90°C under nitrogen.

### 3.2 [^18^F]FPCA

[^18^F]FPCA was produced as per the methodology described by Morris et al*.* 11 In short, 3‐carboxaldehyde‐*N,N,N*‐trimethylpyridine‐2‐aminium bromide (2 mg, 8 μmol) in DMSO (200 μL) was added to the reactor containing azeotropically dried [^18^F]fluoride and heated to 70°C for 10 min. [^18^F]FPCA was trapped and washed on an AFFINIMIP® (AFFINISEP, France) 2.0 mL solid phase extraction (SPE) cartridge. Elution of [^18^F]FPCA was achieved using methanol (3 mL) into the reactor followed by solvent evaporation under vacuum at 60°C for 6.5 min. Synthesis of the 3‐carboxaldehyde‐*N,N,N*‐trimethylpyridine‐2‐aminium bromide precursor is described elsewhere.^11^

### 3.3 [^18^F]FPCA‐F9 solution

A mixture of (Aoa)F9 (1.5 mg, ~880 nmol), anilinium hydrochloride (2.6 mg, 20 μL) and gentisic acid (sodium salt hydrate, 1 mg) in citric acid (0.02 M, 250 μL) was added to purified and dried [^18^F]FPCA (pH 2.7). The reaction mixture was heated to 70°C for 30 minutes and purified using a TSK‐gel® SE‐HPLC column (7.5 mm ID × 30 cm, 10 μm; Hichrom Tosoh Bioscience, Berkshire UK) eluted with PBS with 1% ascorbic acid/1 mL/min, *t*_R_ = 10 minutes and analysed for quality control purposes using size exclusion‐HPLC (SE‐HPLC) (Superdex peptide 10/300 GL, PBS, 1 mL/min, 220 nm, *t*_R_ = 18.05 minutes). The final concentration of the solution was 0.3 mM.

### 3.4 [^18^F]FPCA‐F9 hydrogel

[^18^F]FPCA‐F9 was prepared as described in the previous section. [^18^F]FPCA‐F9 hydrogel was prepared by adding a small amount of [^18^F]FPCA‐F9 to an unlabelled, unfunctionalised F9 hydrogel. In short, unfunctionalised F9 (11.6 mg) in H_2_O (440 μL) was sonicated for 15 minutes before addition of 120 μL of purified [^18^F]FPCA‐F9 in PBS solution. The mixture was vortexed and the pH adjusted to 5.5 using NaOH (0.5 M), and the final volume increased to 600 μL using H_2_O giving a final hydrogel concentration 0.01 M.

## 4 FLUORESCENCE PREPARATION

### 4.1 FITC‐F9 hydrogel

FITC‐F9 hydrogel was prepared using the same procedure as described previously. Unfunctionalised F9 (10.5 mg) in H_2_O (440 μL) was sonicated for 15 minutes before addition of FITC‐labelled F9 (1.2 mg) in DMSO (5 μL). The mixture was sonicated for a further 15 minutes and the pH adjusted to 5.5 using NaOH (0.5 M). The final volume was increased to 491.5 μL using deionised H_2_O giving, once again, a final hydrogel concentration of 0.01 M.

### 4.2 Preclinical analyses

All animal handling was in accordance with UK legislation under the 1986 Animals (Scientific Procedures) Act.

### 4.3 Preclinical PET [^18^F]FPCA‐F9 solution and hydrogel

C3H mice were anaesthetised using isoflurane induction 4% and maintained 1.5% in 70% N_2_O and 30% O_2_ mixture. [^18^F]FPCA‐F9 solution was injected in the tail vein (13 MBq, n = 4) of healthy mice or [^18^F]FPCA‐F9 hydrogel was injected subcutaneously into the right flank of different healthy mice (2‐5 MBq, n = 3). The acquisition protocol parameters consisted of a preliminary CT scan to determine attenuation correction factors followed by a 1‐ and 4‐hour PET acquisition time for [^18^F]FPCA‐F9 solution and hydrogel, respectively. Major excretory organs, including liver, kidney and bladder as well as bone and hydrogel region (in [^18^F]FPCA‐F9 hydrogel scans only), were delineated on CT and uptake was quantified as SUV.

### 4.4 [^18^F]FPCA‐F9 preclinical metabolite analysis

Plasma, kidney, liver and urine samples were taken postsacrifice at 5 (n = 2) and 20 (n = 2) minutes postinjection of [^18^F]FPCA‐F9 monomer. Plasma was extracted from whole blood samples using a Thermo ALC multispeed refrigerated centrifuge PK121R (8720 g, 4°C for 3 minutes). Kidney and liver samples were homogenised and centrifuged (8720 g, 4°C for 3 minutes), and the supernatant was removed for HPLC analysis. Urine samples were directly injected onto HPLC without sample preparation using a GE Superdex peptide 10/300 GL (GE Healthcare, UK) eluted with PBS (1 mL/min, 220 nm).

### 4.5 *In vivo* fluorescence analysis

C3H mice (n = 2) were anaesthetised using isoflurane (induction 4% and maintained 1.5%) in 70% N_2_O and 30% O_2_ mixture. FITC‐F9 gel (200 μL) was injected subcutaneously into the right flank (C3H mice) and fluorescence data attained over 5 days using a 30‐second acquisition time. Relative bioluminescent counts were normalised to acquisition time and quantified in units of counts. Excitation and emission wavelengths were 487 and 547 nm, respectively. FLI integration was 400 ms per frame, and BVR pixel was 171 μm with a pixel size of 170 × 170 μm. Hydrogel site was delineated and emission was quantified as counts per minute.

## 5 RESULTS AND DISCUSSION

### 5.1 [^18^F]FPCA‐F9 radiochemistry

The radiochemical conversion of (Aoa)F9 to [^18^F]FPCA‐F9 by reaction with [^18^F]FPCA was 63% ± 7%, and 400 ± 200 MBq of [^18^F]FPCA‐F9 was produced (decay corrected from end of bombardment) starting with 25 to 30 GBq of [^18^F]fluoride (n = 6). Radiosynthesis of [^18^F]FPCA‐F9 as a solution and a hydrogel was completed within 2 hours from end of bombardment.

Supplementary Figure A provides GE TRACERlab FX‐FN trace for crude reaction mixture. Radiochemical purity of [^18^F]FPCA‐F9 > 98% was attained, as can be seen in Supplementary Figure B. [^18^F]FPCA‐F9 radiochemical yields (RCY) were not reflective of the high radiolabelling efficiency of (Aoa)F9 with [^18^F]FPCA because of significant product loss during the automated radiosynthesis. This was attributable to the agglutinative nature of the peptide and issues of poor solubility and high viscosity of (Aoa)F9, despite using low concentrations; this caused handling difficulties for the automated platform because of the platform's narrow‐bore tubing. Notwithstanding, [^18^F]FPCA‐F9 was produced in sufficient quantities for use in PET analysis. It is anticipated that use of larger‐bore tubing and the ability to transfer the reaction mixture across the platform using a syringe drive, as opposed to a helium flow, which is slow and controlled might improve recovery of [^18^F]FPCA‐F9. However, the uniquely aggregative nature of the hydrogel‐forming peptide precludes its comparison with other [^18^F]FPCA peptide radiolabelling.

### 5.2 [^18^F]FPCA‐F9 solution biodistribution—PET

**Figure 2:**
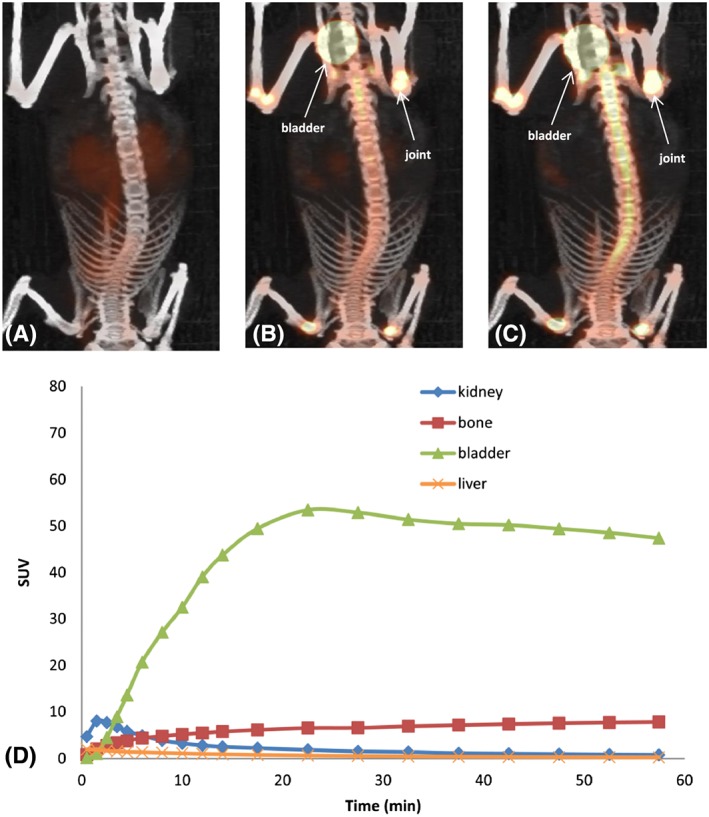
[^18^F]FPCA‐F9 solution at (A) 60 seconds, (B) 15 minutes and (C) 1 hour postinjection and (D) TAC [^18^F]FPCA‐F9 biodistribution in bone and excretory organs (C3H mice, n = 4)

The PET images in Figure 2 A, B and C depict [^18^F]FPCA‐F9 biodistribution at 60 seconds, 15 minutes and 1 hour postinjection, respectively. Figure 2D shows the uptake of [^18^F]FPCA‐F9 over 60 minutes in key organs. Kidney uptake was observed within 5 minutes postinjection (5.9 ± 1.9 SUV).

This was followed by significant bladder accumulation, as clearly seen in Figures 2B and C, which begins to plateau after 20 minutes (53.4 ± 16.6 SUV). Liver uptake remained low over the time course of the scan (1.4 ± 0.1 and 0.7 ± 0.1 SUV at 5 and 20 minutes postinjection, respectively). The concentrations observed within the liver were consistently lower than those seen in the kidney, whereas the highest concentration was seen in the bladder, confirming that the kidney is the major excretory organ for [^18^F]FPCA‐F9 and potential radiometabolite(s). Rapid renal excretion is expected of unmodified, small peptides.^12^, ^13^

Gradual bone accumulation can also be seen (7.8 ± 2.5 SUV 1 hour postinjection), although levels of radioactivity are overshadowed by much higher concentrations in the bladder (47.4 ± 7.2 SUV 1 hour postinjection); bone uptake was most notable at regions of the joint. Accumulation of radioactivity in the bone can be ascribed either to defluorination of [^18^F]FPCA‐F9 or to the interaction of the peptide with the bone tissue. Bone uptake followed kidney uptake, an organ which has been previously implicated in defluorination^14^, ^15^; however, no free [^18^F]fluoride was observed in metabolic analyses (discussed later). We cannot, therefore, exclude the possibility of an interaction between a positively charged [^18^F]FPA‐F9 (at physiological pH) and a negatively charged protein rich cartilage tissue.

This potential interaction is currently being investigated in our laboratory and is outside the scope of this article.

### 5.3 [^18^F]FPCA‐F9 solution metabolite analysis

Liver, kidney, urine and plasma samples were analysed using SE‐HPLC to quantify the degree of metabolic degradation of the peptide. The analysis identified the major peak as [^18^F]FPCA‐F9 with a retention time of ~18 minutes; none of the samples analysed showed any metabolite of [^18^F]FPCA‐F9 (see Supplementary Figure C). Table 1 shows the percentage of parent compound ([^18^F]FPCA‐F9) identified in each of the samples after 60 minutes. The high percentage of parent compound in all samples demonstrates the high stability of [^18^F]FPCA‐F9.

**Table 1:** Quantification of parent ([^18^F]FPCA‐F9) fraction after 60 minutes (data expressed as mean ± SD, n = 4)

Liver	Kidney	Urine	Plasma
94% ± 0%	95% ± 1%	95% ± 1%	100

Liver and kidney samples, though, showed a second peak at ~8 minutes accounting for ~5% of the radioactivity (see Supplementary Figure Cii). Urine samples showed a peak with a retention time of ~12 minutes, accounting for 5% of the radioactivity (see Supplementary Figure Ciii) alongside the [^18^F]FPCA‐F9 peak. A shorter retention time is suggestive of a larger species than [^18^F]FPCA‐F9, conceivably a result of [^18^F]FPCA‐F9 aggregation or interaction with a large molecular weight protein. As discussed above, [^18^F]FPCA‐F9 carries a positive charge under physiological conditions and can therefore interact with negatively charged proteins. The large molecular weight radiolabelled species seen in both liver and kidney samples (*t*_R_, ~8 minutes) falls outside the exclusion limit of the column (*V*_0_, ~9 minutes), therefore suggestive of molecular mass > 12 kDa. The species in the urine sample (*t*_R_, 12.5 minutes) is likely, according to molecular weight standard analysis, to have a molecular mass between 8 and 10 kDa.

The absence of radiolabelled species with a molecular mass smaller than that of [^18^F]FPCA‐F9 and within the 22.5‐minute column exclusion limit is suggestive of the absence of [^18^F]FPCA‐F9 radiometabolites, including [^18^F]fluoride. The absence of a [^18^F]fluoride radiometabolite provides evidence against [^18^F]FPCA‐F9 defluorination and further supports the notions of an interaction between F9 and the cartilage.

If [^18^F]FPCA‐F9 is aggregating with circulating proteins, similar aggregation is expected to be observed in plasma sample, which is not the case. The TAC shows that F9 is rapidly transported to the kidneys, thereby minimising the time for which F9 can interact with circulating proteins such as albumin in the plasma.

Importantly, [^18^F]FPCA‐F9 showed high plasma stability according to metabolite analysis and is rapidly and predominantly excreted by the kidney, thereby showing a favourable biodistribution profile for the use of F9 as a biomaterial.

### 5.4 [^18^F]FPCA‐F9 hydrogel biodistribution—PET

**Figure 3:**
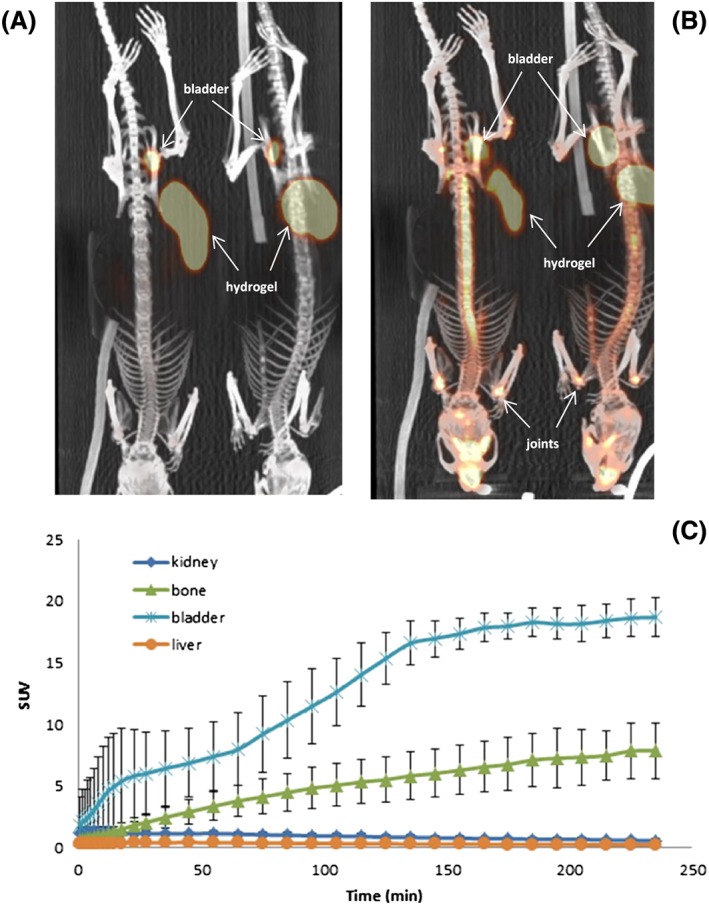
[^18^F]FPCA‐F9 hydrogel at (A) 60 seconds and (B) 4 hours postinjection. (C) TAC showing [^18^F]FPCA‐F9 biodistribution in bone and excretory organs (C3H mice, n = 3)

[^18^F]FPCA‐F9 hydrogel was injected subcutaneously into the flank of C3H mice; its *in vivo* behaviour over a 4‐hour time‐course can be interpreted from Figure 3. Figures 3A and B (60 seconds and 4 hours postinjection) clearly show the radiolabelled hydrogel. Figure 3C provides the biodistribution of [^18^F]FPCA‐F9 peptide in the bone and excretory organs. The increasing concentrations of [^18^F]FPCA‐F9 in the kidneys and bladder clearly show that the peptide is gradually being eluted from the hydrogel as the latter physically degrades/disaggregates.

A progressive increase in radioactivity over 150 minutes is observed in the bladder, before plateauing (17.4 ± 1.3 SUV). Although this is reminiscent of the excretion profile of the peptide solution, albeit over a different time period, there is a 2‐step profile for bladder accumulation that was observed in all mice. This is anticipated to be as a result of the staged nonuniform disaggregation of the hydrogel; smaller and more vulnerable hydrogel fragments at the periphery are expected to disaggregate first and rapidly before the bulk of the hydrogel.

Bone uptake of [^18^F]FPA‐F9 was also observed (3.8 ± 1.3 SUV 1 hour postinjection) in this case and can be seen in Figure 3. Uptake of [^18^F]FPCA‐F9 in the kidney, bladder and liver, major excretory organs (1.15 ± 0.2, 5.9 ± 3.8 and 0.4 ± 0.1 SUV respectively, 20 minutes postinjection), is also shown in Figure 3C. Akin to when [^18^F]FPCA‐F9 was administered as a solution, liver uptake remained below the levels observed in the kidney and significantly below bladder levels, and this was true over the duration of the experiment (0.7 ± 0.1 SUV at 20 minutes postinjection). The work confirms that the peptide exhibited the projected biodistribution with no significant changes in its *in vivo* behaviour, on account of its gelation.

### 5.5 FITC‐F9 hydrogel—fluorescence imaging

Despite the inability to detect FITC‐F9 penetration into the tissue, as a consequence of the limitations imposed by FITC emission, shallow injection of FITC‐F9 produced fluorescence data that were a good indicator of the hydrogel biological half‐life. The intention of using BLI as a complementary technique was to help visualise the process of hydrogel expansion and disaggregation; these data were used to augment PET data, which were used to provide information regarding biodistribution and route of F9 metabolism.

**Figure 4:**
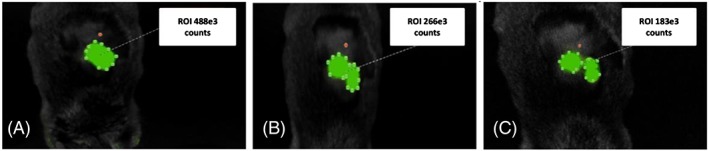
Fluorescence imaging of FITC‐F9 hydrogel (A) 60 seconds, (B) 48 hours and (C) 96 hours postinjection

Fluorescence data are presented in Figure 4; the data show FITC‐F9 hydrogel 60 seconds, 48 and 96 hours postinjection (Figure 4 A, B and C, respectively) in the flank of C3H mice (n = 2).

Qualitative analysis of the preliminary data permits a first appreciation of the results. Signal intensity, corresponding to FITC‐F9, was measured over the course of 5 days, beginning at the time of injection. Steady depletion in signal intensity was observed, with a 43% ± 8% reduction over the first 24 hours and 60 ± 3% over 96 hours, attributable to disaggregation of the hydrogel over time. Results of the fluorescence work, therefore, suggest an F9 hydrogel biological half‐life between 1 and 4 days.

An expansion of ~10% was observed over the first 24 hours, which dropped to ~3% over 48 hours. These results suggest that, postinjection, there is further penetration of the hydrogel in the surrounding tissues. These hydrogels are known to be shear thinning and animal movement will promote gel tissue penetration. This is then followed by the slow disaggregation of the hydrogel.

The fluorescence data, despite not being fully quantitative, are in agreement with PET data providing evidence for the exploitable *in vivo* lifetime of F9 hydrogels as a biomaterial for either tissue engineering or drug delivery applications

## 6 CONCLUSION

We report the automated synthesis of a radiolabelled gel‐forming peptide, [^18^F]FPCA‐F9, and its preclinical PET analysis alongside preliminary fluorescence imaging data; this permitted *in vivo* characterisation of its behaviour. Preclinical results demonstrated renal excretion as the major elimination route for labelled‐F9 monomer, both after administration as a solution and a hydrogel. Results also showed the high plasma stability of the labelled‐F9 monomers and established a favourable biological half‐life of labelled‐F9 hydrogel. The current work has confirmed that the behaviour exhibited by F9 is typical of natural, small peptides,^12^ and its *in vivo* biodistribution is not altered on account of its gel form.

Collectively, the fluorescence and PET data provide insight into a possible *in vivo* pathway for F9 hydrogel, first characterised by hydrogel tissue penetration then followed by disaggregation and final elimination via renal excretion. Because of the natural amino acid composition of F9 alongside its *in vivo* metabolic profile, F9 is not expected to have issues of toxicity.^16^ Such characteristics would highlight F9 hydrogel as ideal biomaterial that could be used as a silent carrier for localised drug delivery. This is of particular importance in oncological applications wherein targeted drug delivery helps to ensure maximal drug dose at the tumour while minimising drug delivery to nontarget sites.

## DISCLOSURE STATEMENT

The authors declare no potential sources of conflict of interest or competing financial interest.

## Supporting information

Figure S1.
TRACERlab FX‐FN gamma trace of crude reaction mixture [^18^F]FPCA‐F9
