# Supplementary material for: In vivo characterisation of a therapeutically relevant self‐assembling 18F‐labelled β‐sheet forming peptide and its hydrogel using positron emission tomography
Source: J Labelled Comp Radiopharm. 2017 Aug 4;60(10):481–8. doi: 10.1002/jlcr.3534 (PMC5601235; doi:10.1002/jlcr.3534)
Supplement: Supplementary file 1 — Figure S1. TRACERlab FX‐FN gamma trace of crude reaction mixture [18F]FPCA‐F9 [file JLCR-60-481-s001.docx]

# Supplementary

*Figure A: TRACERlab FX-FN gamma trace of crude reaction mixture [^18^F]FPCA-F9*

[^18^F]FPCA-F9

[^18^F]FPCA

*Figure Bi and ii: UV and radio-chromatogram of [^18^F]FPCA-F9, respectively*

(Aoa)F9

[^18^F]FPCA-F9

F

i

ii

*Figure Ci: Radio-chromatogram showing plasma metabolite sample*

Counts

*Figure Cii: Radio-chromatogram showing liver metabolite sample*

Counts

*Figure Ciii: Radio-chromatogram showing urine metabolite sample*

Counts
